# Supplementary material for: Baseline Absolute Lymphocyte Count and ECOG Performance Score Are Associated with Survival in Advanced Non-Small Cell Lung Cancer Undergoing PD-1/PD-L1 Blockade
Source: J Clin Med. 2019 Jul 10;8(7):1014. doi: 10.3390/jcm8071014 (PMC6678702; doi:10.3390/jcm8071014)
Supplement: Supplementary file 1 [file jcm-08-01014-s001.pdf]

Table S1. Optimal quantile cut-offs for dichotomization of patients for NLR, ALC, and ANC based on maximal Harrel's C-index.

	NLR					ALC					ANC				
QUANTILE [%]	CUTOFF NLR	N1 (NLR_LO)	N2 (NLR_HI)	C-INDEX_PFS	C-INDEX_OS	CUTOFF ALC	N1 (ALC_LO)	N2 (ALC_HI)	CIND_PFS	C-INDEX_OS	CUTOFF ANC	N1 (ANC_LO)	N2 (ANC_HI)	CIND_PFS	C-INDEX_OS
26	3.00	39	102	0.5339	0.5879	0.874	37	104	0.5488	0.5944	4.094	37	104	0.4856	0.5467
27	3.00	39	102	0.5339	0.5879	0.896	38	103	0.5499	0.5986	4.164	38	103	0.4815	0.5519
28	3.12	40	101	0.5300	0.5778	0.902	40	101	0.5509	0.6095	4.222	40	101	0.5280	0.5603
29	3.20	42	99	0.5337	0.5894	**0.928**	41	100	0.5591	0.6210	4.276	41	100	0.4768	0.5617
30	3.30	46	95	0.5293	0.5845	0.950	44	97	0.5485	0.6071	4.390	44	97	0.4764	0.5644
31	3.30	46	95	0.5293	0.5845	0.954	44	97	0.5485	0.6071	4.396	44	97	0.4764	0.5644
32	3.30	46	95	0.5293	0.5845	0.976	45	96	0.5563	0.6056	4.449	45	96	0.4733	0.5636
33	3.40	49	92	0.5432	0.5995	0.984	47	94	0.5540	0.5999	4.552	47	94	0.4711	0.5722
34	3.40	49	92	0.5432	0.5995	1.006	48	93	0.5596	0.6010	4.566	48	93	0.4767	0.5711
35	3.50	51	90	0.5414	0.6045	1.010	51	90	0.5515	0.6137	4.570	50	91	0.4771	0.5648
36	3.54	51	90	0.5414	0.6045	1.018	51	90	0.5515	0.6137	4.638	51	90	0.5291	0.5710
37	3.60	54	87	0.5419	0.6087	1.038	52	89	0.5488	0.6102	4.750	52	89	0.5352	0.5764
38	3.62	54	87	0.5419	0.6087	1.050	56	85	0.5514	0.6091	4.782	54	87	0.5228	0.5789
39	3.70	56	85	0.5501	0.6087	1.050	56	85	0.5514	0.6091	**4.827**	55	86	0.5247	0.5802
40	**3.80**	59	82	0.5558	0.6219	1.060	58	83	0.5506	0.6057	4.850	57	84	0.4840	0.5632
41	3.80	59	82	0.5558	0.6219	1.064	58	83	0.5506	0.6057	5.024	58	83	0.4849	0.5675
42	3.88	59	82	0.5558	0.6219	1.070	60	81	0.5534	0.6068	5.068	59	82	0.4800	0.5705
43	4.00	62	79	0.5509	0.6149	1.092	61	80	0.5512	0.6035	5.112	61	80	0.4865	0.5557

Individual selected cut-offs are indicated as bold (maximal C-indices are yellow). N1 and N2 are number of patients in the two dichotomized groups.

Table S2. Comparison of baseline characteristics between anti-VEGF exposed and anti-VEGF naïve advanced NSCLC patients receiving PD-1/PD-L1 blockade.

	No Prior/Concomitant Anti-VEGF Therapy N = 125	Prior/Concomitant Anti-VEGF Therapy * N = 17	*P*-value
**Median age** (range)	67 (26–89)	63 (50–76)	0.068 ‡
**Sex**			0.336
male	73 (58%)	12 (71%)	
female	52 (42%)	5 (29%)	
**ECOG performance status**			0.424
0	33 (27%)	6 (35%)	
1	75 (60%)	11 (65%)	
2	14 (11%)	0 (0%)	
3	3 (2%)	0 (0%)	
**Histology**			0.002
non-squamous	79 (63%)	17 (100%)	
squamous	46 (37%)	0 (0%)	
**Smoking history**			0.546
smoker	103 (87%)	13 (93%)	
never-smoker	15 (13%)	1 (7%)	
missing	7 (6%)	3 (18%)	
**TNM stage**			0.131
III	15 (12%)	0 (0%)	
IV	110 (88%)	17 (100%)	
**ALK translocation**			0.519
no	115 (98%)	16 (100%)	
yes	3 (2%)	0 (0%)	
missing	7 (6%)	1 (6%)	
**EGFR mutation status**			0.830
wild-type	114 (93%)	16 (94%)	
mutant	9 (7%)	1 (6%)	
missing	2 (2%)	0 (0%)	
**CNS involvement**			0.708
no	98 (78%)	14 (82%)	
yes	27 (22%)	3 (18%)	
**PD-L1 status**			0.024
positive	70 (67%)	5 (36%)	
negative	35 (33%)	9 (64%)	
missing	20 (16%)	3 (18%)	
**PD-L1 status category**			0.052
<1%	35 (34%)	9 (64%)	
1-50%	35 (34%)	4 (29%)	
>50%	34 (32%)	1 (7%)	
**ICB therapy line**			0.002
1st line	38 (30%)	2 (12%)	
2nd line	62 (50%)	5 (29%)	
≥ 3rd line	25 (20%)	10 (59%)	
**Immune-checkpoint inhibitor**			0.215

nivolumab	67 (54%)	12 (71%)	
pembrolizumab	49 (39%)	3 (17%)	
atezolizumab	9 (7%)	2 (12%)	
**Tertiary oncologic center**			
Salzburg	44 (35%)	6 (35%)	0.994
Linz	81 (65%)	11 (65%)	
**Prior/concomitant denosumab application**			
no	93 (74%)	13 (77%)	0.854
yes	32 (26%)	4 (23%)	
**Prior radiotherapy**[#]			
no	72 (58%)	7 (41%)	0.201
yes	53 (42%)	10 (59%)	
**Subsequent therapy**			
no therapy	77 (62%)	8 (47%)	
taxane-based	17 (14%)	2 (12%)	0.555
TKI	14 (11%)	3 (18%)	
other	17 (14%)	4 (23%)	
**Antibiotic treatment during ICB**[§]			
no	69 (55%)	11 (65%)	0.458
yes	56 (45%)	6 (35%)	

ECOG: Eastern Cooperative Oncology Group, EGFR: epidermal growth factor receptor, ALK: Anaplastic lymphoma kinase, PD-L1: programmed cell death ligand 1, ICB: immune-checkpoint blockade, VEGF: vascular endothelial growth factor, TKI: tyrosine kinase inhibitor. [‡] two-sided Wilcoxon rank-sum test, [*] bevacizumab, ramucirumab or nintedanib, [§] administration of antibiotics within a time frame of one month before or one month after initiation of immune-checkpoint blockade, [#] to the primary tumor or metastases.

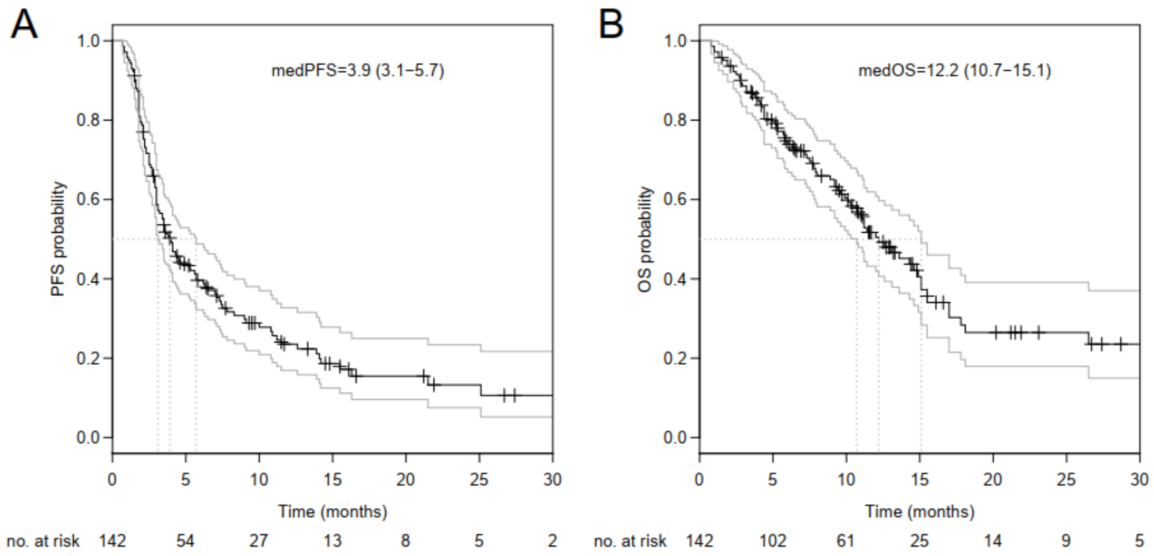

Figure S1. Kaplan-Meier curves for PFS (**A**) and OS (**B**) from initiation of PD-1/PD-L1 blockade in 142 advanced NSCLC patients. medPFS is median progression free survival and medOS is median overall survival; 95% confidence interval in brackets.

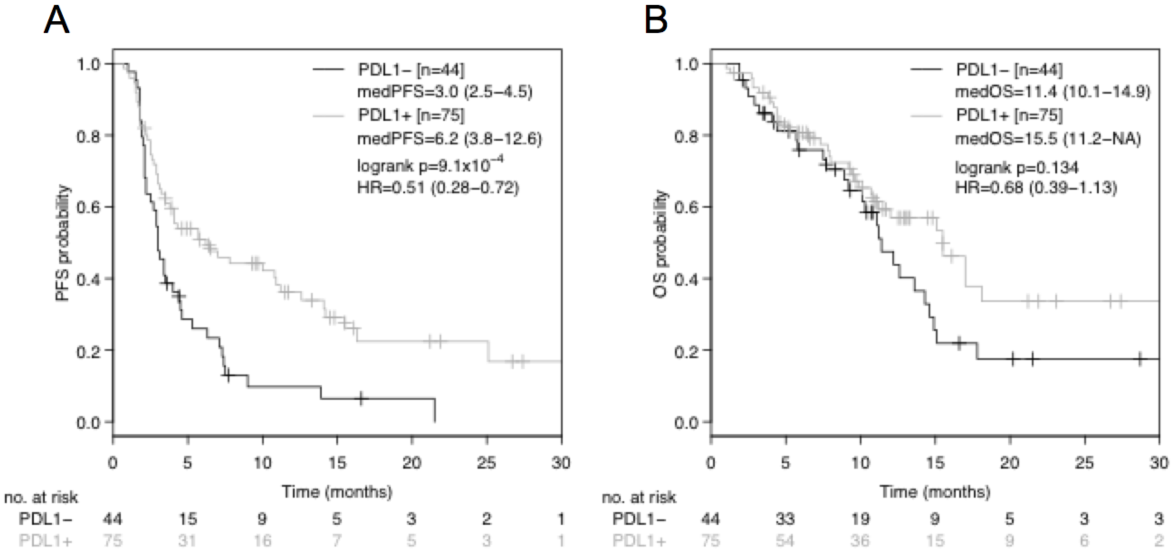

Figure S2. Kaplan-Meier curves for PFS and OS according to PD-L1 expression status on tumor cells. Comparison of Kaplan-Meier curves for PFS (**A**) and OS (**B**) between PD-L1+ and PD-L1- advanced NSCLC groups. HR is hazard ratio, 95% confidence interval in brackets.

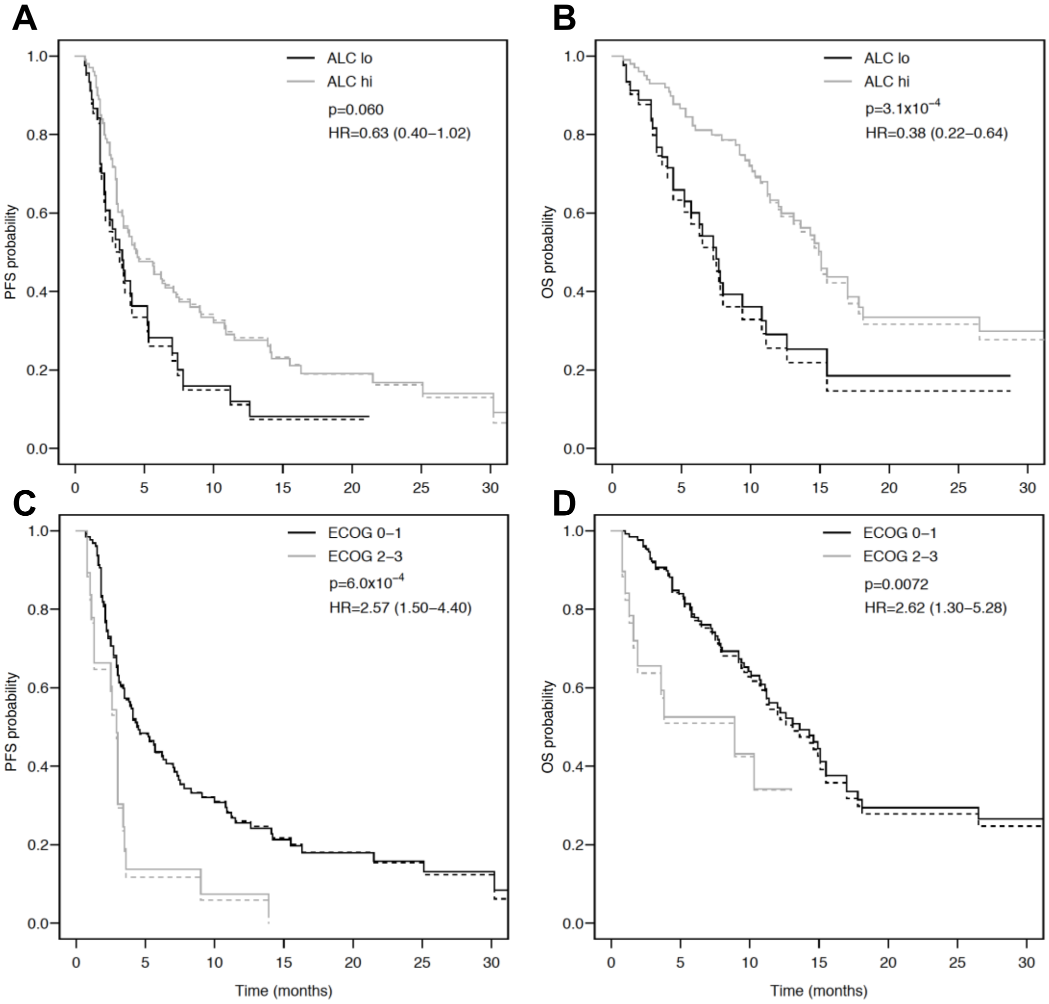

Figure S3. Therapy line adjusted survival curves for PFS and OS according to absolute lymphocyte count and ECOG performance status. Comparison of survival curves in advanced NSCLC patients with a baseline ALC >0.93x10⁹/L versus ≤0.93x10⁹/L for PFS (**A**) and OS (**B**), and with a baseline ECOG

performance status >1 versus ≤1 for PFS (**C**) and OS (**D**). dotted lines: original; solid lines: adjusted for therapy-line (1+2 versus ≥3). HR is hazard ratio, 95% confidence interval in brackets.

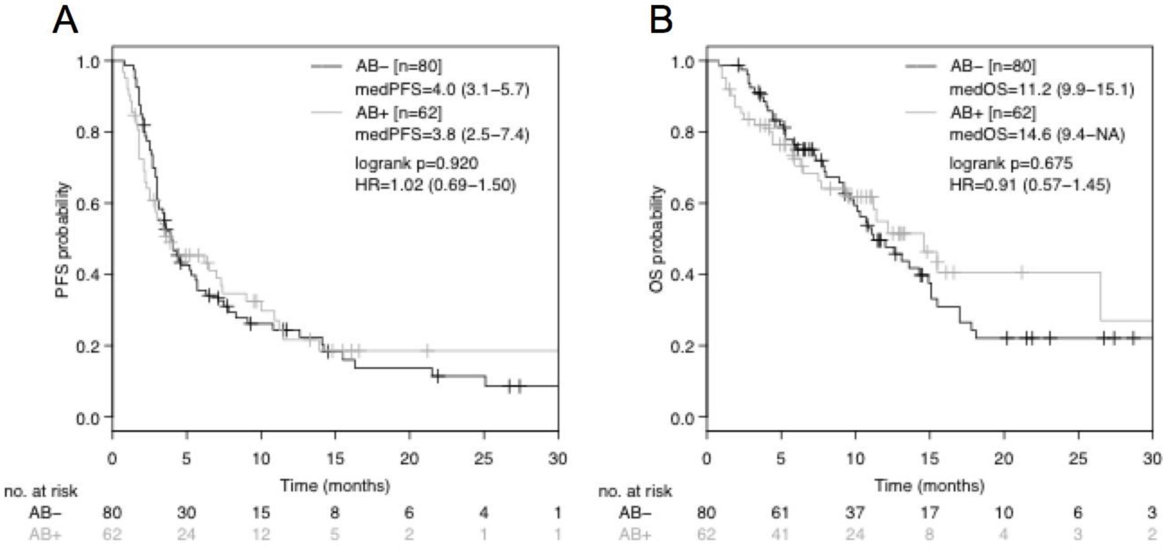

Figure S4. Kaplan-Meier curves for PFS and OS according to antibiotic treatment status. Comparison of Kaplan-Meier curves for PFS (**A**) and OS (**B**) between antibiotic-positive and antibiotic-negative group in advanced NSCLC. Antibiotic exposure in temporal proximity to immune-checkpoint inhibitor therapy start was defined as antibiotic therapy administration within one month before or one month after initiation of ICB. HR is hazard ratio, 95% confidence interval in brackets.
